# Microscopic Observation Drug Susceptibility (MODS) Assay: A Convenient Method for Determining Antibiogram of Clinical Isolates of *Mycobacterium tuberculosis* in Ghana

**DOI:** 10.3390/medsci8010005

**Published:** 2020-01-25

**Authors:** Enid Owusu, Mercy Jemima Newman

**Affiliations:** 1 Department of Medical Laboratory Sciences, School of Biomedical and Allied Health Sciences, University of Ghana, P.O. Box LG 25, Accra, Ghana; 2 Department of Medical Microbiology, University of Ghana Medical School, University of Ghana, P.O. Box LG 25, Accra, Ghana; mjnewmangh@gmail.com

**Keywords:** MODS, *Mycobacterium tuberculosis*, susceptibility, tuberculosis

## Abstract

(1) Background: Present methods for drug susceptibility tests (DST) rely on culture methods that are sophisticated and relatively faster, or a slow and cheaper option. These methods frustrate disease control; therefore, there is a need for methods that incorporate key functions of microscopy and culture, with reduced cost burden and sophistry. Thus, the purpose of this study was to identify which, among the most commonly used (in Ghana) methods, can conveniently be used at health centers located in rural areas for effective DST determination of *Mycobacterium tuberculosis* (*MTB*). (2) Methods: *Mycobacterium tuberculosis* isolates were tested for their susceptibility to streptomycin, isoniazid, rifampicin, ethambutol (SIRE), and pyrazinamide by microscopic observation drug susceptibility (MODS) and BACTEC MGIT 960 methods. Evaluations were based on shorter turnaround periods, rapidity, ease of use, cost, etc. A comparative analysis was statistically expressed as kappa values. (3) Results: Endpoints for drug susceptibilities by MODS averaged 13 days (7–32), whilst that for BACTEC MGIT 960 was 10 days with a further 12 days to detect resistance. Therefore, a turnaround period of 22 days was needed for DST by BACTEC MGIT 960, compared to 13 days for MODS. There were differences in correlation levels between the two methods, as determined by their kappa values. (4) Conclusion: The MODS assay was found to be less costly, more user-friendly, and still able to be conveniently used at health centers located in rural areas known to be endemic for TB, particularly in Ghana.

## 1. Introduction

Pulmonary tuberculosis (TB) is a preventable disease with a huge global impact due to its mode of transmission. It remains one of the world’s leading causes of death, killing more than 2 million people every year [1,2,3]. According to the 2019 WHO Global Tuberculosis Report, geographically, most TB cases in 2018 were in the WHO regions of South-East Asia (44%), followed by Africa, with 24% [4]. Earlier reports have indicated that more than 95% of cases and deaths due to TB occur in developing countries [3,5]. Therefore, the prevalence of the condition is reportedly relatively high in countries with limited resources, such as Ghana [3,5]. This problem could be attributed to the poor hygienic practices, unfavorable living conditions, and resource constraints in these areas. Comorbidity with HIV coupled with reports of emerging resistant TB strains (MDR-TB) has frustrated efforts at disease control [5,6,7]. Rapid detection of the disease coupled with faster methods for the determination of susceptibility to *Mycobacterium tuberculosis* (*MTB)* isolates to primary treatment antibiotics is a major step in efforts to control the disease. Various microbiological methods are available for the susceptibility determination of *MTB* to treatment with drugs/antibiotics, such as streptomycin, rifampicin, ethambutol, isoniazid, and pyrazinamide [8,9]. These drugs have been used effectively for several decades; however, the emergence of *MTB* strains showing varying levels of resistance to the drugs has frustrated efforts at TB control, a situation that is particularly associated with endemic countries with limited resources. This phenomenon is present in Ghana [5], where drug-resistant TB continues to pose a public health threat. In 2018, there were about half a million new cases of rifampicin-resistant TB (of which 78% had multidrug-resistant TB) [4].

Methods for determining drug susceptibility tests (DST) for *MTB* involve the use of an isolate culture in a drug-incorporated mycobacterial medium. Frequently used media include Lowenstein-Jensen (L-J) egg based, Middlebrook 7H11 solid media, and Middlebrook 7H9 liquid medium. Methods for DST determination include the automated BACTEC MGIT 960 broth culture system and the Lowenstein-Jensen solid culture assay. Among these culture methods, the automated BACTEC assay is the most sensitive; this approach is suitable for growing *MTB* even in AFB negative samples within relatively shorter detection periods (7–14 days) [10], though it is sophisticated and costly. The burden of cost makes this method inaccessible to peripheral healthcare points in TB endemic communities [10]. The L-J solid culture method is relatively simpler and cheaper, but it is slow, requiring longer culture periods (4–8 weeks) for DST determination. The period can be shortened with the use of Middlebrook and Cohn’s solid media using agar [11]; however, its long turnaround time makes it unreliable, in addition to this media not being easily available in some locations. In the midst of these challenges, new cases of tuberculosis continue to be reported annually [4]. This situation, coupled with the emergence of MDR-TB, as well as comorbidity with infections such as HIV, tend to aggravate the situation [5,6,7].

The need for drug susceptibility methods that are cheap, rapid, convenient, and easily applicable in rural health posts of TB-endemic areas cannot be overemphasized. Such methods will provide useful information on suitable antibiotics and their effective therapeutic doses, as well as on the current prevalence of circulating MDR-TB in endemic areas. These efforts will contribute immensely to effective disease control. As emphasized by some researchers, a viable method is one that would incorporate functionally-suitable aspects of microscopy and culture, and also consider robustness, while being technically less demanding [12,13,14].

The microscopic observation drug susceptibility (MODS) assay is one such qualitative test which fits into these criteria. It involves the visualization of *MTB* growth in broth, in addition to typical cord (pellicle) formation. This method has been recommended by several studies as being relatively rapid, cost effective, and less technically demanding [15,16,17,18,19]. Its associated limitations have to do with interspecies differentiation, a challenge which can easily be overlooked, given the advantages derived from the rapid results obtained from this method [14]. The adaptation of the MODS assay can be helpful for the early determination of DST for TB, particularly in under-resourced areas. The sensitivity of the MODS method has been estimated to be about 94%, while that of Lowenstein-Jensen (LJ) culture has been reported to be about 86.9% [15].

Though many tools exist for the diagnosis of *MTB* disease, they are limited by their inability to provide results on the status of drug resistance [16,17,18,19,20,21,22,23]. This study therefore purposes to identify which, among the most commonly used (in Ghana) methods, can conveniently be applied at health centers located in rural areas for effective DST determination of *MTB* isolates. Thus, the MODS and BACTEC MGIT 960 methods were evaluated for their usefulness in DST determination of *MTB,* using clinical *MTB* isolates. Specifically, the study sought to evaluate the feasibility of applying these methods in resource-constrained areas by evaluating their turnaround periods, rapidity, ease of use, and cost effectiveness.

## 2. Materials and Methods

### 2.1. Study Design, Setting and Subjects

This was a cross-sectional study conducted at the chest clinic of the Korle-Bu teaching hospital, the premier public tertiary hospital and the largest referral hospital in Ghana. The study was carried out in a Class II biosafety cabinet in a biosafety level 3 (BSL3) laboratory. All safety precautions were appropriately observed. Subjects who consented to participate in the study were instructed on sputum collection, as recommended by the World Health Organization (WHO) [24]. Each patient was required to produce three sputum samples for the investigation, i.e., one on the spot, an early morning sample the next day, and a third one upon arrival at the laboratory. Some of the study participants could not produce all three samples, while samples which contained saliva instead of sputum were excluded. Thus, a total of 533 sputum samples were obtained from 221 clinically-suspected *MTB* patients who consented to be enrolled in the study. After initial screening using Zeihl-Neelsen smear microscopy, 130 samples with smears showing no acid fast bacilli (*MTB*) were excluded. Therefore, a total of 403 samples qualified to be used for the study. Out of the 403 samples, 54 participants decided to opt out of the study. Thus, a total of 349 samples were finally available for analyses. Ethical approval was given by the Scientific and Ethical review boards of Ministry of Health and the Korle-Bu Teaching hospital.

### 2.2. Microbiological Analysis of Study Samples

A minimum of one (1) and a maximum of three (3) sputum samples were obtained from each subject. These were processed to reduce fast growing bacteria contaminants by the *N*-acetyl-l-cysteine decontamination method [25]. The decontaminated sputum samples were then routinely analyzed by the Zeihl-Neelsen smear microscopy, and cultured on LJ media [26], as well as the MODS assay [14] and the BACTEC MGIT 960 (Becton Dickinson, Sparks, MD, USA) method [27] for *MTB* growth.

### 2.3. Mycobacterium Tuberculosis Culture

Sputum samples were decontaminated by the N-A-L-C-sodium hydroxide method as described in [25], pelleted by centrifugation (at 3,200× *g* for 20 min), and resuspended in 1000 µL of sterile saline. The turbidity of the suspension was adjusted to a McFarland number 1 standard and then used to inoculate for cultures, as well as DST by BACTEC MGIT 960 and direct MODS methods. The isolated cultures from processed samples were then used for the indirect MODS DST method. The procedures used were adapted from Caviedes et al. [17] and Musa et al. [28].

For culture on LJ media, 250 μL of decontaminated bacterial suspension was inoculated. The LJ slants were incubated at 37 °C and examined twice a week for 42 days. Growth on the LJ slants was checked with the Zeihl-Neelsen smear microscopy (Sigma-Aldrich, St. Louis, MO, USA) for the presence of AFB. Growth was recorded at 28 days and at 42 days with the following descriptions: +++ for confluent growth, ++ for >100 colonies, and 1–100 actual numbers of colonies.

For culture with *BACTEC MGIT 960*, 500 μL of bacterial suspension was inoculated in MGIT tubes and incubated at 37 °C in the *BACTEC MGIT 960* system, which monitors for fluorescence every day for up to 42 days by automated mechanisms.

With the MODS culture method, 130 μL of decontaminated bacterial suspension was inoculated in *Middlebook* 7H9 broth and incubated at 37 °C for 42 days. The wells were examined daily after day five under an inverted microscope for growth; bacterial growth was seen as granular serpentine cords. All negative cultures were further confirmed with Z-N microscopy after the 42 days’ incubation.

### 2.4. Drug Susceptibility Testing (DST)

Drug susceptibility tests were performed by the *BACTEC MGIT 960* susceptibility method, as well as the direct and indirect MODS methods. The procedures for *BACTEC MGIT 960,* as well as the direct and indirect MODS methods, are briefly described below.

#### 2.4.1. BACTEC MGIT 960 Susceptibility Method

Drug susceptibility tests set up by the BACTEC system required five tubes for the test drugs and two others for pyrazinamide (PZA). Streptomycin, isoniazid, rifampicin, and ethambutol (SIRE) were prepared in dilutions of 1:100 (1%), whilst pyrazinamide (PZA) was diluted to 1:10 (10%). Control tubes contained broth without drugs. Each test contained 7 mL of broth with 800 µL of the supplement and 100 µL of specified antibiotic. Then, 500 µL of *MTB* inoculum was dispensed into each test tube. Control tubes contained no antibiotics, whiles 1:100 inoculum dilutions were set up as microbial controls; this was to ensure growth in the controls before tests. DST was performed for all positive MGIT on Isoniazid 0.2 μg/mL, Streptomycin 4 μg/mL, Rifampicin 40 μg/mL, Ethambutol 2 μg/mL, and Pyrazinamide 200 μg/mL, according to WHO guidelines [29].

Tests and controls were arranged in the BACTEC MGIT 960 instrument for a 14-day incubation period. Tests with growth units of less than 200 were classified as indeterminate, and therefore, repeated. Controls usually attained 400 growth units before flagging for end point. Tests with fewer than 100 growth units were indicative of susceptibility. Smears were made from controls and Z-N stained to establish purity.

#### 2.4.2. Microscopic Observation Drug Susceptibility (MODS)

The MODS susceptibility assay was conducted by the direct and indirect methods. The tests were done in a 12-well culture plates. Each test well contained 2 mL Middlebrook 7H9 broth, 200 µL of suspension, and 34 µL of the appropriate test antibiotic. A McFarland 0.5 (approximately 104 CFU/mL) suspension of isolates was used. The controls were set up in the first row of the plate. For each plate, two antibiotics in combinations of streptomycin and isoniazid (S&I) and rifampicin and ethambutol (R&E) were set up with the control. The drug concentrations were similar for the BACTEC MGIT 960 method according to WHO guidelines [29].

Each plate was set up with a complement of a positive and a negative control in addition to the test wells. Tests for pyrazinamide were set up on a separate plate, as this required conditions that were different from those of the other test drugs, including a relatively low pH and so a higher dose of inoculum. Each PZA test well contained 2 mL of Middle brook 7H9 broth, 200 µL of supplement, and 34 µl of reconstituted PZA. All tests and controls were appropriately labeled.

Tests for the direct method were inoculated with 140 µL of decontaminated sputum sample prepared in pelleted dilutions as indicated. An indirect test was inoculated with appropriate diluents of *MTB* colony suspension. A negative control well contained only MODS media. A positive control contained the MODS media and *MTB* inoculum at a dilution of 1:100 and 1:10 for PZA.

Plates were incubated at 37 °C and examined twice weekly after an initial daily check for contamination for up to 42 days. Detectable serpentine clusters (pellicle formation) of *MTB* growth observed under an inverted microscope at 40× magnification and confirmed by AFB microscopy was indicative of positive culture. The growth observed in both the drug-free and the drug-containing wells was indicative of resistance, whilst tests with growth in drug-free and no growth in drug-incorporated media indicated susceptibility of the test isolates to the test drugs [18]. If growth was detected in only one control well, MODS-DST was recorded as indeterminate for technical analysis

The broth from the MGIT tubes was poured into the MODS wells to find out whether cord formation in acidic medium could be easily visualized. The broth medium for the determination of DST for streptomycin, isoniazid rifampicin, and ethambutol (SIRE) was prepared in the laboratory. With respect to pyrazinamide testing, the quality could have been compromised if the media had been prepared in the laboratory.

### 2.5. Statistical Analysis

Data obtained from this study was stored and analyzed using the Microsoft office Excel 2007 software (Microsoft^®^ Office Professional 2007, Microsoft Corporation, Redmond, WA, USA) and GraphPad software version 8.0. (GraphPad, La Jolla, CA, USA). The concordance of susceptibility results was determined based on the sensitivity, specificity, and positive and negative predictive values for the detection of resistance with 95% confidence intervals (CIs), as well as with kappa values [30]. A kappa coefficient (κ) value of <0.4, 0.4–0.6, 0.61–0.8, and 0.81–1.0 indicated low agreement, moderate agreement, substantial agreement, and perfect agreement, respectively. A *p*-value less than 0.01 was considered statistically significant. For the estimations of the sensitivity, specificity, and predictive value of detection for the methods, a positive reference result was defined as a positive culture according to at least one method for which cross contamination had been conclusively ruled out. A negative reference result was defined as any sample in which all three culture methods yielded negative results, or when two were negative and the third indeterminate, owing to repeated bacterial or fungal overgrowth as defined in an earlier study [14].

## 3. Results

### 3.1. Drug Susceptibility Testing

A total of 349 samples were analyzed. Two hundred and eighty three samples were tested for susceptibility to SIRE and 66 to pyrazinamide by the MODS method. Seventeen (17) samples were lost after the MODS method, thus, two hundred and sixty six samples were tested for susceptibility to streptomycin, isoniazid, rifampicin and ethambutol (SIRE) and 66 to pyrazinamide, all by the MGIT method. The average duration from culture to drug susceptibilities by MODS was 12.5 (approximately 13 days, range of 7–32) for the direct method and 9.3 days (approximately 9 days, range of 6–16) for the indirect method, whilst for BACTEC MGIT 960, it was 10.1 days for culture (approximately 10 days, range 3–40), with a further 12 days to detect resistance (Table 1). Therefore, a turnaround period of 22 days was needed for DST by BACTEC MGIT 960, compared to 13 days for MODS. The difference between the turnaround time for DST by the two methods (BACTEC MGIT 960 and MODS) was statistically significant (*p* < 0.01).

Table 1 shows the time taken for the reading of susceptibility of *MTB* isolates to tested antimicrobials by the various methods.

### 3.2. Resistance Pattern

Growth in the tubes or wells containing any of the antimicrobials was interpreted as a sign of resistance to that particular antimicrobial. Susceptible samples were those that showed no growth in the antimicrobial tubes or wells, even though there was growth in the corresponding control test tube or well. True resistance was defined as resistance by the two methods, and true susceptibility as those that were susceptible to the two methods. Indeterminate represents samples in which that the MGIT instrument flagged positive but no AFB was seen in their smears; thus, a possible contamination of culture was considered to have occurred, or growth was detected in only one control well for MODS-DST. The sensitivity, specificity, and predictive values of all the antimicrobials are displayed in Table 2.

Tested *MTB* isolates resistance to the primary antimicrobials by the MODS method were streptomycin (27.9%), isoniazid (27.9%), rifampin (8.1%), ethambutol (8.1%), and pyrazinamide (6.1%). Meanwhile, those for the MGIT method were streptomycin (19.6%), isoniazid (24.5%), rifampicin (7.2%), ethambutol (4.2 %), and pyrazinamide (7.6%) (Table 2). Sensitivity of the MODS method with respect to streptomycin was 100%, isoniazid (5.7%), rifampicin (7.2%), ethambutol (85.2%), and pyrazinamide (100%), while sensitivity of the BACTEC MGIT 960 method with respect to streptomycin was 81.3%, rifampicin (85.2%), and pyrazinamide (100%) (see Table 2).

Concordance between MODS and MGIT for the individual antimicrobials as calculated by kappa coefficients (κ) were streptomycin (0.87), isoniazid (0.79), rifampin (0.90), ethambutol (0.63), and pyrazinamide (1.0). Kappa coefficients values that were excellent (perfect agreement) were observed for streptomycin, rifampin, and pyrazinamide (0.87–1.0) across the two tested methods, giving a nearly completely concordant result. However, low substantial agreement was apparent for ethambutol. Table 3 presents concordance and discordance of the MODS (direct and indirect) and BACTEC MGIT 960 methods.

## 4. Discussion

The current study purposed to identify which, among the most commonly used (in Ghana) methods, can conveniently be applied at health centers located in rural areas for effective DST determination of *MTB* isolates. Therefore, the MODS and BACTEC MGIT 960 methods were evaluated for their usefulness in DST determination of *MTB,* using clinical *MTB* isolates. This specifically involved a comparison of the drug susceptibility outcome for MODS to BACTEC MGIT 960, and of the ease of recognizing cord formation using each of the two methods.

The study determined the susceptibility of *MTB* isolates to streptomycin, rifampicin, isoniazid, ethambutol, and pyrazinamide, the primary antimicrobials for TB treatment by the BACTEC MGIT method, as well as by the MODS method. Tested *MTB* isolates showed varying resistance and susceptibility to the primary antimicrobials by the MODS (6.1–27.9 and 68.2–93.9 respectively) and MGIT (4.2–24.5 and 61.9–86.4 respectively) methods. Meanwhile, the concordance between MODS and MGIT with respect to streptomycin was 0.87, isoniazid (0.79), rifampin (0.90), ethambutol (0.63), and pyrazinamide (1.0).

There were, however, a few discrepancies in the results of the two methods with respect to ethambutol and, to a lesser extent, isoniazid (correlation between the two methods was 63% for ethambutol and 79% for isoniazid); however, these discrepancies were not enough to make the results obtained by the two methods statistically different. The correlation between the two methods for rifampin (90%) and isoniazid (79%) was in accordance with a previous work which compared MODS to the proportion method [18]. Other researchers [31,32] have found the correlation between MODS and other methods to be poor with regard to ethambutol and streptomycin.

In contrast, this study recorded levels that were higher. All the same, the extent of disagreement was not statistically significant [18]. Another study also observed disagreements between the results for rifampin and those of MODS in comparison with other methods [33]. In that study, the results were read 14 days after visible growth appeared in the drug free wells [32]. There was an earlier suggestion by Caviedes et al. [17] that the results for rifampin resistance should be confirmed with either Microplate Alamar Blue Assay (MABA) or the proportion method on Lowenstein-Jensen media.

In the current study, however, the observed differences in the results of MGIT and MODS could be attributed to any of the following three factors: inoculum size, method of reading the results, or failure rates with respect to the MGIT method. These reasons are interrelated. MGIT variation in bacterial concentration could be reduced, since the results were not manually read. On a given day of positivity, the bacterial concentrations are usually fairly the same. A manual reading of MODS makes it subjective, indicating that the concentration on the day of positivity may not be the same. Unlike the MGIT, that measures the unit of growth as well, there are no discrete colonies to count in the case of MODS, and therefore, any growth in a drug-containing well was deemed to reflect resistance to that drug. The high number of indeterminate cases (13.6%) with respect to the MGIT method could account for the differences observed in the results of susceptibility testing by the two methods. 

Regarding resistance, other studies have reported varying level of resistance in different parts of Ghana [5,31,33]. A comparative analysis of the results obtained by these studies revealed that the percentage of resistance observed in the current study was relatively higher for most of the tested antibiotics. For Streptomycin, while the current study recorded resistance of 27.9%, previous studies have observed 0%, 9%, and 23% [5,31,33]. The resistance levels for the other antibiotics tested were as follows: Isoniazid: 27.9% compared to 4.2% and 23% [4,31], Rifampin: 8.1% compared to 0% and 0.7% [5,33], Ethambutol: 8.1% compared to 0% [5,33], and Pyrazinamide: 6.1% compared to 0% and 0.5% [5,33]. MDR-TB was also recorded to be 6.7% for the current study, compared to 0% and 2.2% in earlier studies [5,33]. It is noteworthy that the current study observed resistance in the two methods for more antimicrobials (6), compared to previous studies by other investigators in Ghana [5,31,33].

The marked increase in resistance to virtually all the primary antimicrobials within the study period represents a major health concern. For instance, the isoniazid resistance of 27.9% observed in this study could have negative implications for TB patients, since such resistance is associated with poor clinical outcomes [34], with recent guidelines already having been issued for a global rollout of an isoniazid-resistant regimen [35]. Increases in resistance have been recorded at other locations near Ghana. N’Guessan et al. [36] reported higher prevalence of multidrug-resistant tuberculosis in Côte d’Ivoire from 1995 to 2016. It has also been shown in a systematic review on the prevalence of DR-TB in Nigeria and Ethiopia that the current WHO estimate for new and previously-treated TB patients for these countries was low [37,38], providing evidence that the burden of DR-TB in high-TB-burden countries, especially in Africa, is probably higher than what has been predicted [4].

Considering the individual antimicrobials separately, there were rises in resistance to isoniazid (5.0 to 6.9%) and rifampicin (1.0 to 1.2%), but not to ethambutol and pyrazinamide. In Belarus, out of 163 TB isolates that were tested against the antibiotics, 42 (68.5%) were identified as being resistant to isoniazid, rifampicin, and streptomycin, and eight (28%) were resistant to ethambutol [34]. Monoresistance to isoniazid was observed in four (14%) isolates [39]. In this study, the reading of cord formation via the MODS method was done with ease. This is an important criterion; therefore, the MODS method is hereby endorsed, as it is less technically challenging, and hence, can easily be used in a clinical setting with limited facilities, especially in rural parts of in Ghana.

## 5. Conclusions

This study determined that the MODS method is efficient in determining the susceptibility of *MTB* isolates to primary antibiotics for TB treatment. This method was observed to be cost effective and easily applicable in health centers of TB-endemic, rural communities in Ghana. It could therefore be introduced alongside the BACTEC MGIT system, with little training being required due to its ease of use.

## Figures and Tables

**Table 1:** Time taken (in days) for culture and susceptibility testing.

	Culture and Susceptibility Testing (MGIT, Direct and Indirect MODS)						
	MGIT Culture, *N* = 283	MGIT SIRE, *N* = 266	MGIT PZA, *N* = 66	MGIT Susceptibility, *N* = 266	Dir. MODS SIRE, *N* = 283	In. MODS SIRE, *N* = 283	MODS PZA, *N* = 66
Maximum	40	21	21	63	32	16	16
Minimum	3	5	5	10	7	6	7
Average	10.1	9.6	9.0	21.7	12.5	9.3	9 *p* < 0.01
Indeterminate	13	36	4	NA	0	0	0

*N* = Total number of *MTB* isolates tested; SIRE-streptomycin, isoniazid, rifampicin, ethambutol; PZA-pyrazinamide; In. MODS-Indirect MODS; Dir MODS-Direct MODS; Indeterminate represents samples that the MGIT instrument flagged as positive but in which no AFB was seen in their smears; thus, a possible contamination of culture was considered to have occurred, or growth was detected in only one control well for MODS-DST. Tests and controls were arranged in the BACTEC MGIT 960 instrument for a 14-day incubation period. MGIT susceptibility represents the turnaround time for the BACTEC MGIT 960 procedure (from culture through drug susceptibility testing). As part of the MGIT test protocol, about two days on average elapsed between the day the instrument flagged positive and the day the susceptibility was set.

**Table 2:** Resistance pattern of the antimicrobials and sensitivity, specificity, and predictive values.

**MGIT (For SIRE, *N* = 266 and for PZA, *N* = 66)**					
	**Streptomycin**	**Isoniazid**	**Rifampicin**	**Ethambutol**	**Pyrazinamide**
Resistant, *n* (%)	52 (19.6)	65 (24.5)	19 (7.2)	11 (4.2)	5 (7.6)
Susceptible, *n* (%)	177 (66.8)	164 (61.9)	210 (79.2)	218 (82.3)	57 (86.4)
Indeterminate, *n* (%)	36(13.6)	36 (13.6)	36 (13.6)	36 (13.6)	4 (6.0)
Sensitivity (%)	81.3	82.3	79.2	57.9	100
Specificity (%)	100	97.6	91.3	99.1	100
PPV (%)	100	94.2	86.4	84.6	100
NPV (%)	93.7	92.1	97.7	94.5	100
**MODS (for SIRE, *N* = 283 and for PZA, *N* = 66)**					
	**Streptomycin**	**Isoniazid**	**Rifampicin**	**Ethambutol**	**Pyrazinamide**
Resistant, *n* (%)	79(27.9)	79(27.9)	23(8.1)	23(8.1)	4(6.1)
Susceptible, *n* (%)	204 (72.1)	193 (68.2)	260 (91.9)	260 (91.9)	62 (93.9)
Indeterminate, *n* (%)	0	0	0	0	0
Sensitivity (%)	100	95.7	85.2	85.2	100
Specificity (%)	94	93.2	97.4	97	100
PPV (%)	91	86.5	76.7	74.2	100
NPV (%)	100	98.0	98.5	99.2	100

*N* = Total number of *MTB* isolates tested; SIRE-streptomycin, isoniazid, rifampicin, ethambutol; PZA-pyrazinamide; PZA-Pyrazinamide; PPV denotes a positive predictive value and NPV a negative predictive value of the n-number of samples that were observed to be resistant, susceptible, or indeterminate.

**Table 3:** Concordance and discordance of the MODS (direct and indirect) and BACTEC MGIT 960 methods.

	Antimicrobials				
	Rifampin	Streptomycin	Isoniazid	Ethambutol	Pyrazinamide
**Concordance (%)**	90	87	79	63	100
**Discordance (%)**	10	13	21	37	0
